# Antagonistic roles of tau and MAP6 in regulating neuronal development

**DOI:** 10.1242/jcs.261966

**Published:** 2024-10-07

**Authors:** Xiaohuan Sun, Wenqian Yu, Peter W. Baas, Kazuhito Toyooka, Liang Qiang

**Affiliations:** Department of Neurobiology and Anatomy, Drexel University College of Medicine, Philadelphia, PA 19129, USA

**Keywords:** Tau, MAP6, Microtubule, Axon growth, Growth cone turning, Neuronal migration

## Abstract

Association of tau (encoded by *Mapt*) with microtubules causes them to be labile, whereas association of MAP6 with microtubules causes them to be stable. As axons differentiate and grow, tau and MAP6 segregate from one another on individual microtubules, resulting in the formation of stable and labile domains. The functional significance of the yin–yang relationship between tau and MAP6 remains speculative, with one idea being that such a relationship assists in balancing morphological stability with plasticity. Here, using primary rodent neuronal cultures, we show that tau depletion has opposite effects compared to MAP6 depletion on the rate of neuronal development, the efficiency of growth cone turning, and the number of neuronal processes and axonal branches. Opposite effects to those seen with tau depletion were also observed on the rate of neuronal migration, in an *in vivo* assay, when MAP6 was depleted. When tau and MAP6 were depleted together from neuronal cultures, the morphological phenotypes negated one another. Although tau and MAP6 are multifunctional proteins, our results suggest that the observed effects on neuronal development are likely due to their opposite roles in regulating microtubule stability.

## Teaser

**Highlighted Article:** Tau and MAP6 play antagonistic roles in regulating multiple aspects of neuronal development, presumably via their antagonistic effects on microtubule dynamics.

## INTRODUCTION

Neurons depend on microtubules (MTs) for their exaggerated morphologies and cargo transport needs (Kapitein and Hoogenraad, 2015; Baas et al., 2016). Throughout all stages in the life of the neuron and in all neuronal compartments, the MT array consists of a labile fraction and a stable fraction, the former of which is highly dynamic and the latter of which is far less dynamic (Mitchison and Kirschner, 1984; Baas and Black, 1990; Baas et al., 2016). The terms stable and labile were coined on the basis of how rapidly or slowly the MTs in each fraction depolymerize in response to drugs, cold or dilution (Baas et al., 2016). The stable fraction is important for preserving the architecture of the neuron, whereas the labile fraction permits morphological plasticity (Baas et al., 2016). The stable and labile MT fractions derive from the binding to the MT of various MT-associated proteins (MAPs) with different on–off rates for association and dissociation.

In the axon, the stable and labile fractions comprise two distinct domains on individual MTs, with the stable domain toward the minus end of the MT and the labile domain toward the plus end. Each domain can become quite long, with intermittent stabilization of the labile domain or portions of it enabling the stable domain to grow longer (Baas et al., 2016). The labile domain of MTs extending into the distal axon and growth cone is especially labile compared to the labile domains of MTs that exist along the shaft of the axon (Stepanova et al., 2010; Dent and Kalil, 2001). In other compartments of the neuron, the stable MT fraction is comparatively less stable than in the axon, and the MT domains on individual MTs are less distinct (Baas et al., 2016). In dendrites, the two fractions might also segregate spatially, with MTs that are more stable forming centralized bundles, surrounded by MTs that are less stable (Kapitein and Hoogenraad, 2015; Katrukha et al., 2021). One question that arises is how neurons are able to preserve a robust labile MT fraction in the face of proteins that are so effective at stabilizing the stable fraction.

Tau (encoded by *Mapt*) is a MAP known for influencing the properties of MTs in the axon (Arendt et al., 2016; Wang and Mandelkow, 2016; Mueller et al., 2021), with long-standing dogma implicating tau as a MT stabilizer (Dehmelt and Halpain, 2004; Kolarova et al., 2012; Kadavath et al., 2015). This view was challenged by our recent studies on cultured rodent neurons showing that reducing tau levels by RNA interference does not result in destabilization of axonal MTs but rather in the selective loss of the labile MT fraction (Qiang et al., 2018). Additional work has shown that the remainder of the labile fraction became less labile (i.e. more stable) when tau was depleted owing to increased binding to MTs of another MAP, namely MAP6. MAP6, which has bona fide MT-stabilizing properties, is enriched on the stable domains of axonal MTs (Slaughter and Black, 2003; Cuveillier et al., 2021). Taken together, these findings suggest that tau enables the assembly of long labile domains of MTs in the axon and prevents them from being stabilized by MAP6, at least until a signaling pathways recalibrates the balance between these two MAPs to allow MT stabilization to occur.

Our previous studies lacked functional tests of the significance of the antagonistic relationship between these two MAPs in regulating MT stability. This is not as easy question to address because each of the two MAPs has functions other than influencing MT stability (Lefèvre et al., 2013; Gory-Fauré et al., 2014; Tortosa et al., 2017; Cuveillier et al., 2020, 2021; Mueller et al., 2021; Bloom and Baas, 2024). However, if depletion of one produces a phenotype opposite to depletion of the other, such effects would likely be due to their opposite effects on MT stability. In this Short Report, we have sought to test this prediction in three different experimental systems.

## RESULTS AND DISCUSSION

The two MT fractions (i.e. the labile and stable domains), can vary in how stable or labile they are and this is reflected quantitatively in the levels of tubulin in each fraction that are tyrosinated or post-translationally detyrosinated (Baas et al., 2016; Qiang et al., 2018; Baas and Qiang, 2019). In experiments on primary cultures of rat neurons, the labile domain of axonal MTs labels highly for tyrosinated tubulin whereas the stable domain shows little or no labeling in analyses using either immune-electron or immunofluorescence microscopy (Baas and Black, 1990; Brown et al., 1993). The labile domains of MTs extending into the distal region of the axon are especially labile, and correspondingly, their levels of tyrosinated tubulin are even higher than in the labile domains along the axon shaft (Ahmad et al., 1993). Thus, the levels of tyrosinated tubulin serve as a quantitative marker/proxy for how labile the MTs are.

**Fig. 1.**
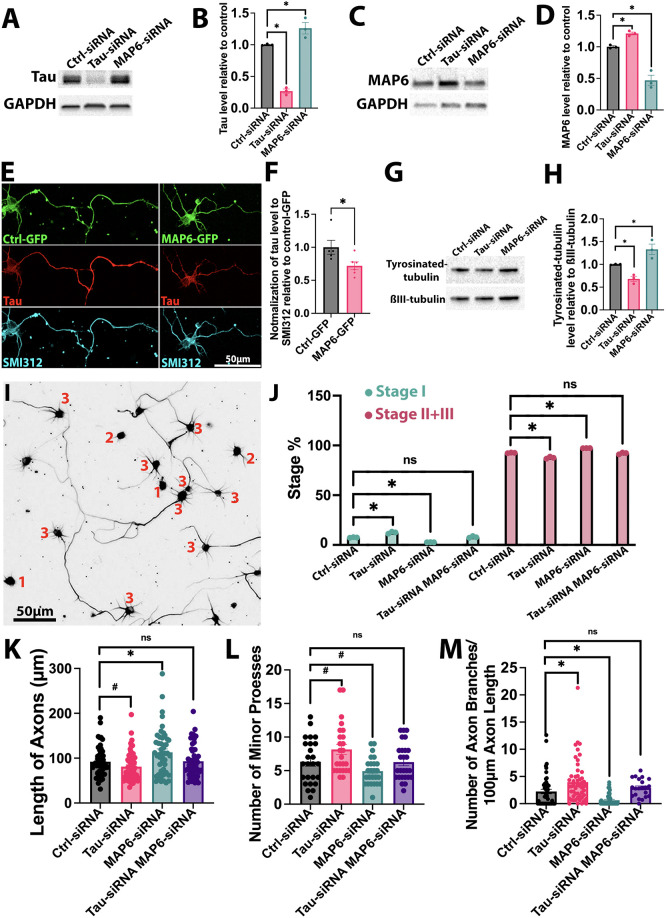
**Opposite effects of depletion of tau or MAP6 on various aspects of MT dynamics and neuronal development in rat hippocampal neurons.** (A–D) Western blots of tau, MAP6 and GAPDH in control, tau siRNA, and MAP6 siRNA-treated neurons (A,C). Tau siRNA decreased tau by 73.22±3.39% and increased MAP6 by 26.05±9.01% (B,D). MAP6 siRNA decreased MAP6 by 53.07%±0.80% and increased tau by 21.04±2.32% (*n*=3). *P<0.05 (one-way ANOVA with Dunnett's multiple comparisons test). (E) Images of neurons transfected with control GFP and MAP6–GFP, showing GFP (green), tau (red) and SMI321 (Cyan). (F) The ratio of tau level to SMI312 level, relative to the control, was measured. Bar graph shows that MAP6 overexpression reduced tau by 28.03±7.26% (*n*=5 for control; *n*=6 for MAP6–GFP). *P<0.05 (two-tailed unpaired *t*-test). (G) Western blots of tyrosinated tubulin and βIII-tubulin in control, tau siRNA, and MAP6 siRNA neurons. (H) Tau siRNA decreased tyrosinated tubulin by 31.88±5.70%, whereas MAP6 siRNA increased it by 33.01±11.66% (*n*=3). *P<0.05 (one-way ANOVA with Dunnett's multiple comparisons test). (I) Example image of neurons stained for βIII tubulin in stages I, II, and III. (J) Tau siRNA increased the proportion of stage I neurons by 4.90±0.55%, MAP6 siRNA decreased them by 4.76±0.05% (*n*=3). *P<0.05; ns, not significant (one-way ANOVA with Dunnett's multiple comparisons test). (K–M) Axon length, minor processes and axon branches per 100 µm in control, tau siRNA, MAP6 siRNA and dual siRNA-treated neurons. (K) Tau siRNA shortened axons by 10.97±4.12 µm (*n*=60), MAP6 siRNA lengthened them by 21.01±7.54 µm (*n*=50). *P<0.05; ns, not significant (one-way ANOVA with Dunnett's multiple comparisons test); ^#^*P*<0.05 (two-tailed unpaired *t*-test). (L) Tau siRNA increased minor processes by 1.87±0.74, MAP6 siRNA decreased them by 1.4±0.40 (*n*=25). ^#^*P*<0.05; ns, not significant (two-tailed unpaired *t*-test). (M) Tau siRNA increased axon branches by 1.74±0.54 (*n*=52), MAP6 siRNA decreased them by 1.6±0.15 (*n*=51). *P<0.05; ns, not significant (one-way ANOVA with Dunnett's multiple comparisons test). All quantitative results are mean±s.e.m.

In hippocampal neurons 4 days after introduction of siRNA, we found via western blotting that knocking down tau by 73.22% led to a 26.05% increase in MAP6 levels, whereas knocking down MAP6 by 53.07% resulted in a 21.04% increase in tau levels (Fig. 1A–D). Correspondingly, transfecting GFP-tagged MAP6 plasmids (Deloulme et al., 2015) for 2 days into hippocampal neurons reduced tau levels by 28.03% compared to the neurons transfected with control GFP plasmid, normalized by use of the axonal marker SMI312 antibody (Fig. 1E,F). These results suggest that the levels of tau and MAP6 in the neuron are interdependent and responsive to changes in the levels of one another. After confirming the effectiveness of tau and MAP6 siRNA, we then examined tyrosinated tubulin levels as an indicator of decreased or increased MT stability (Qiang et al., 2018; Peris et al., 2022) and found a 31.88% decrease in tyrosinated tubulin levels when tau was knocked down. Conversely, a 33.01% increase in tyrosinated tubulin levels was observed in MAP6-depleted neurons compared to controls (Fig. 1G,H).

Next, we explored the impact of experimental tau or MAP6 reductions on the morphology of hippocampal neurons during their development. These neurons were cultured for 2 days after introduction of the relevant siRNA and then replated and cultured for an additional 2 days. We examined neuronal morphology by categorizing neurons based on their developmental stages (Fig. 1I), following the previously published classification (Dotti et al., 1988). Briefly, in stage I, neurons exhibit a lack of processes and maintain a rounded shape encircled by lamellipodia. Moving to stage II, neurons begin to develop a few minor processes around the cell body, and in stage III, one of these minor processes extends to become the axon. Our results show that 4.9% more tau knockdown neurons stayed in stage I, with 4.9% fewer neurons achieving stage II and III compared to control neurons, indicating that the reduction of tau slightly slows neuronal development compared to hippocampal neurons treated with the control siRNA (Fig. 1I,J). By contrast, MAP6-depleted neurons display the opposite phenotype, with accelerated neuronal development, showing that 4.76% more tau knockdown neurons achieved stage II and III, whereas 4.76% fewer neurons stayed in stage I compared to control neurons (Fig. 1I,J). These findings indicate that tau reduction in cultured hippocampal neurons delays their stepwise development, whereas MAP6 reduction accelerates it. Strikingly, no significant difference was found in neuronal developmental stages between control siRNA-treated neurons and those in which both tau and MAP6 were simultaneously knocked down via siRNA (Fig. 1I,J).

Finally, we evaluated the length of the axon, the number of minor processes and the number of axonal branches per 100 µm in stage III neurons. Compared to the control, tau-depleted neurons developed shorter axons by 10.97 µm with more minor processes by 1.87 per soma (Fig. 1K,L). In contrast, MAP6-depleted neurons extended longer axons by 21.01 µm with fewer minor processes by 1.4 per soma, compared to control neurons (Fig. 1K,L). Indeed, tau-depleted neurons had greater numbers of axonal branches by 1.74 per 100 µm in axonal length, compared to control neurons; by contrast, MAP6- depleted neurons had fewer axonal branches by 1.6 per 100 µm, compared to control (Fig. 1M). When tau and MAP6 were simultaneously knocked down via siRNA, no differences in axon lengths, the numbers of minor processes, or the numbers of axonal branches when compared to the control siRNA treated neurons (Fig. 1K–M).

**Fig. 2.**
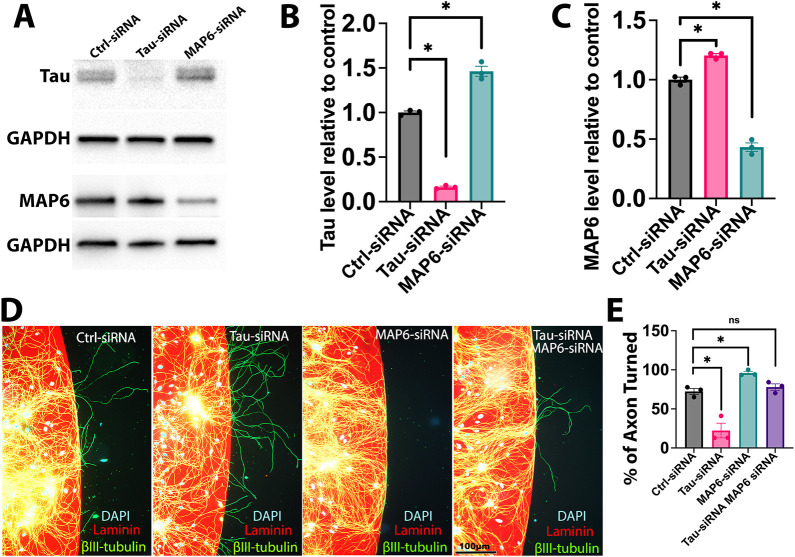
**Opposite effects of depletion of tau or MAP6 on growth cone turning of cultured rat SCG neurons.** (A–C) Western blots of tau, MAP6 and GAPDH in control, tau siRNA- and MAP6 siRNA-treated neurons. Tau siRNA decreased tau by 83.69±0.93% and increased MAP6 by 20.44±1.35%. MAP6 siRNA decreased MAP6 by 56.74±3.12% and increased tau by 46.21±4.91% (*n*=3). *P<0.05 (one-way ANOVA with Dunnett's multiple comparisons test). (D) Example images of neurons with control, tau siRNA, MAP6 siRNA, or both siRNAs, showing DAPI (blue), laminin (red) and βIII-tubulin (green). Neurons on the left are growing on a laminin-coated surface, while their axons extend towards the right, where they encounter a poly-D-lysine surface without laminin coating. (E) Percentage of axon turning at the laminin–poly-D-lysine border. Tau siRNA reduced turning by 50.03±9.39%, and MAP6 siRNA increased it by 23.49±2.08% compared to control (72.37±3.78%). Dual siRNA neurons showed 77.91±4.12% turning (*n*=3). *P<0.05; ns, not significant (one-way ANOVA with Dunnett's multiple comparisons test). All quantitative results are mean±s.e.m.

Domains of most MTs that extend into the distal axon and growth cone are predominantly labile, rich in tau and deficient in MAP6. We previously established that depletion of tau causes these MT domains to become stable and rich in MAP6, whereas depletion of MAP6 causes these MT domains to become even more labile or depolymerize (Qiang et al., 2018). This was done on the basis of nocodazole sensitivity, which is an even better readout of stability than tyrosinated tubulin staining. To investigate the influence of tau and MAP6 on the dynamic behaviors of the growth cone, we carried out experiments using a previously established laminin and poly-D-lysine border assay. For these studies, we used superior cervical ganglion (SCG) neurons because they were previously used with this assay (Nadar et al., 2008; Liu et al., 2010), and because we wished to test our hypothesis about tau and MAP6 in different kinds of neurons. In this assay, these neurons were cultured for 2 days after nucleofection with the corresponding siRNA on a platform coated with poly-D-lysine, with laminin added to the medium. They were then replated and cultured for an additional 2 days on the same platform, with one-half coated with poly-D-lysine and the other half coated with a laminin matrix layered on top of the poly-D-lysine. The laminin-coated section is the preferred growth environment, whereas the other half lacks laminin, creating an environment that is much less preferred. As their axons extend and reach the laminin–poly-D-lysine border, owing to their inclination to remain on the laminin side, most growth cones guide the axons to turn and persist on the laminin-coated region. This process requires labile domains of MTs extending into the distal segment of the axon and requires them to be even more labile than the labile domains in the axon shaft (Buck and Zheng, 2002; Suter et al., 2004). Strikingly, when tau levels were reduced by 83.69%, we observed a 20.44% increase in MAP6 level (Fig. 2A–C) and 50.03% decrease in the amount of SCG axons turning towards the laminin border, compared to control neurons (Fig. 2D,E). By contrast, when MAP6 was reduced by 56.74% (Fig. 2A–C), we observed a 46.21% increase in tau level and 23.49% increase in the amount of SCG axons turning towards the laminin border, compared to control neurons (Fig. 2D,E). Notably, neurons with simultaneous knockdown of tau and MAP6 through siRNA were indistinguishable from control neurons with regard to growth cone turning (Fig. 2D,E).

**Fig. 3.**
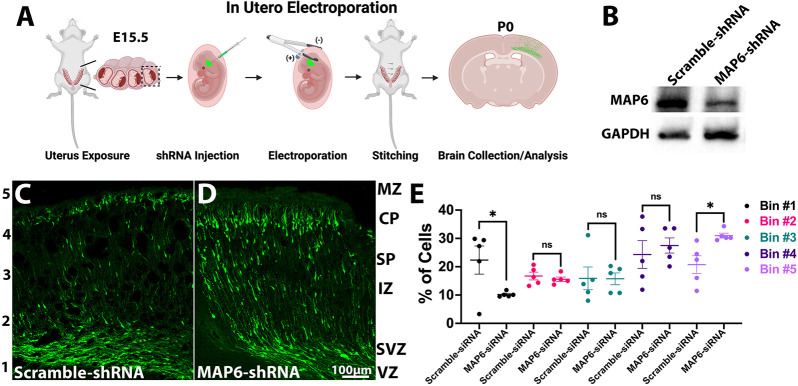
**Acceleration of neuronal migration by MAP6 depletion.** (A) Schematic of *in utero* electroporation. (B) Western blots of MAP6 and GAPDH in scramble and MAP6 shRNA-transfected brains. Images representative of three repeats. (C,D) Embryos injected with scramble (C) or MAP6 shRNA (D) showing transfected neurons (green) in the cortex divided into 5 bins. (E) Percentage of neurons per bin. MAP6 shRNA reduced neurons in bin #1 by 12.15±4.12% and increased them in bin #5 by 10.24±8.61% compared to scramble (*n*=5). *P<0.05; ns, not significant (two-tailed unpaired *t*-test). All quantitative results are mean±s.e.m.

**Fig. 4.**
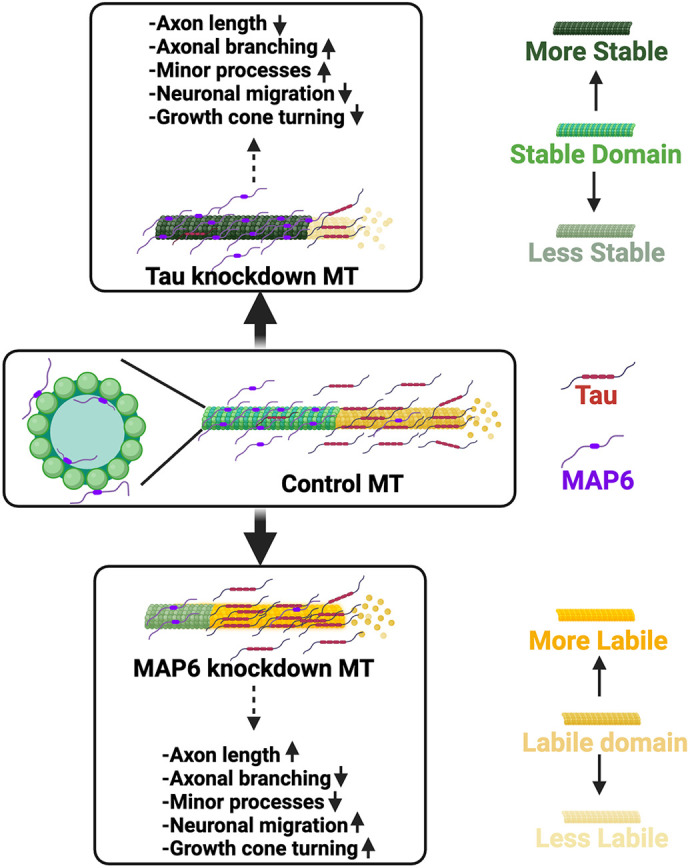
**Diagram of how tau and MAP6 are posited to affect aspects of neuronal development and function via their antagonistic impacts on MT dynamics.** Axonal MTs consist of two domains, a stable domain and a labile domain, distinguished by their dynamic properties. Tau predominantly binds to the labile domain, whereas MAP6 binds to the stable domain, both of which help maintain the structural and functional integrity of axonal MTs. When tau is depleted, the loss of the labile domain leads to MT destabilization, while the stable domain becomes overly stable. This results in shorter axonal length, increased axon branching and minor processes, and delayed neuronal migration and impaired growth cone turning. By contrast, when MAP6 is depleted, MTs lose their stable domains, causing the labile domain to become overly dynamic. This manifests as increased axonal length, reduced axon branching and minor processes, along with accelerated neuronal migration and enhanced growth cone turning. For further details, see main text.

Cortical neurons generated in the ventricular zone (VZ) migrate to their destinations in an inside-out fashion (Stiles and Jernigan, 2010). The migration process involves leading process extension, nucleus translocation and tail process retraction (Lambert de Rouvroit and Goffinet, 2001). Previous studies have demonstrated that tau reduction through shRNA results in tardiness in migrating neurons reaching their destinations (Sapir et al., 2012). To investigate whether MAP6 has an inverse effect on neuronal migration, we conducted a similar experiment by injecting MAP6 shRNA into mouse embryos at embryonic day (E)15.5 (Fig. 3A). At postnatal day 0 (P0), the brains were collected for fixation and analysis. The effectiveness of MAP6 knockdown was verified (Fig. 3B). Then, we conducted an analysis of neuronal migration speed by dividing the cortical region into five bins of equal size. Subsequently, we examined the percentage of neurons within each bin. A higher concentration of neurons in bin #5 denotes faster migration. Conversely, a larger population of neurons remaining in bin #1 suggests slower migration. Our results show that successfully transfected neurons whose MAP6 levels are reduced were labeled with Venus. 10.24% more neurons transfected with MAP6 shRNA migrated to bin #5 compared to scramble shRNA-transfected neurons. Additionally, 12.15% more scramble shRNA-transfected neurons remained in bin #1 compared to MAP6 shRNA-transfected neurons. The outcomes indicate that MAP6 depletion accelerates neuronal migration (Fig. 3C–E). By contrast, when tau is depleted via electroporation, neuronal migration is inhibited compared to that seen in control mice (Sapir et al., 2012). This indicates that tau and MAP6 have inverse roles in regulating neuronal migration (Fig. 4).

### Closing remarks

The expanding knowledge of MAPs, such as their multiple functions and wide interactomes, demands caution in interpreting phenotypes of knockdown experiments. For example, tau has been implicated in synaptic plasticity (Robbins et al., 2021), gene expression (Montalbano et al., 2021), signal transduction (Mueller et al., 2021) and mitochondria homeostasis (Eckert et al., 2014), whereas MAP6 plays diverse roles in interacting with actin filaments (Peris et al., 2018), membranes (Cuveillier et al., 2020) and neuroreceptors (Cuveillier et al., 2021). However, given that tau and MAP6 knockdown have opposite effects on MT stability as well as opposite effects on these morphological parameters, it seems likely that the observed yin–yang effects are attributable to MT stability. This conclusion is fortified by the finding that, with regard to most parameters studied, knocking down both tau and MAP6 together results in preservation of the control phenotypes.

It is unlikely that tau and MAP6 compete for the same MT-binding site because they have very different MT-binding motifs (Cuveillier et al., 2021). In fact, some recent studies suggest that MAP6 might bind inside the lumen of the MTs and induce apertures in the tubulin lattice in a manner quite distinct from tau (Cuveillier et al., 2020). A potential explanation for the competition between these two MAPs might lie in a recent study showing that the binding of a particular MAP changes the MT lattice to make it more or less amenable to the binding of more of the same MAP or other MAPs (Monroy et al., 2018). Indeed, tau exhibits positive cooperativity in its binding to the MT lattice, wherein the initial tau–MT association induces a conformational change in the lattice. This structural alteration enhances the subsequent binding of tau to the lattice, establishing a positive feedback loop in the tau–MT interaction (Monroy et al., 2018; Siahaan et al., 2022) that might inhibit MAP6 from binding to the MTs. It seems reasonable, based on evidence to date, that tau is usually the better competitor, with MT stabilization occurring only when MAP6 is allowed to outcompete tau, for example through phosphorylation events that decrease the MT-binding affinity of tau.

## MATERIALS AND METHODS

### Animals

The use of all animals was in accordance with the guidelines set by NIH and the Drexel University IACUC.

### Rat embryonic hippocampal neuronal cultures

The hippocampus was dissected from TP18-day Sprague Dawley rat fetuses of either sex using cold dissection medium L-15 (Gibco, 21083027). Subsequently, the entire hippocampus was collected and cut into small pieces using microscissors. These pieces were then incubated with 0.25% trypsin (Gibco, 15090046) and 0.4 mg/ml DNase (Thermo Fisher Scientific, EN0521) in a 37°C water bath for 15 min. After incubation, the tissue was washed three times with full plating medium, consisting of neurobasal medium (Gibco, 21103049), 2% B27 supplement (Gibco, A3582801), 0.297% glucose (Sigma-Aldrich, G8769), 1% GlutaMAX-I (Gibco, 35050061) and 10% fetal bovine serum (Novus Biologicals, S11150-NOV), with each wash lasting 5 min. Gentle trituration was performed to separate any clumps, and the neurons were then counted using a hemocytometer (Thermo Fisher Scientific, 0267110). The neurons were cultured for 2 days and then replated for an additional 2 days of culture.

### SCG neuronal cultures

SCGs obtained from P0 to P3 Sprague Dawley rat pups of both sexes and cultured with established protocols (Muralidharan and Baas, 2019). Prior to plating, targeted siRNA, as described below, was introduced into SCG neurons through nucleofection using the Nucleofector 2b system from Lonza. After nucleofection, the cells were cultured for a period of 48 h on 35-mm-diameter culture dishes coated with 0.1 mg/ml poly-D-lysine (Sigma-Aldrich, P0899-50MG). Subsequently, the cells were transferred to glass-bottomed dishes (Cellvis, D35-14-1.5-N), which were also coated with 0.1 mg/ml poly-D-lysine, and a 30 µl droplet of laminin (Invitrogen, 23017-015) was applied. 50,000 SCG neurons were replated on to each dish, and strategically placed in the region where the laminin droplet had been deposited. On day 4, these neurons were fixed and subjected to immunostaining for βIII-tubulin and laminin, and staining with DAPI, as described below.

### RNAi-based knockdown of tau and MAP6

Small interfering RNA (siRNA) targeting scramble, tau or MAP6 was introduced into rat hippocampal or SCG neurons using a Nucleofector (Amaxa), and the manufacturer's G-13 program. The tau or MAP6 siRNA consisted of a mixture of three distinct siRNA duplexes designed to target various regions of each molecule, acquired from Sigma's custom siRNA service. These are the same sequences verified in our previous study in which we conducted appropriate controls, including rescue experiments (Qiang et al., 2018). A nonspecific duplex III, previously utilized, served as the control. The siRNA was prepared at a concentration of 200 nM, aliquoted and subsequently stored at −20°C. The final siRNA concentration utilized for transfection was 10 nM. After transfection, neurons were cultured for 48 h on plastic dishes coated with either poly-L-lysine (Sigma-Aldrich, P2636-25MG) for hippocampal neurons or poly-D-lysine for SCG neurons before undergoing replating. The siRNA-treated neurons were then maintained for 2 days prior to fixation.

### MAP6 overexpression in hippocampal neurons

50,000 hippocampal neurons were cultured for 2 days and then transfected with either 1.5 µg of control-GFP tagged plasmid or MAP6–GFP-tagged plasmid (both a gift from Dr Annie Andrieux, Université Grenoble Alpes, France) using Lipofectamine 2000 (Invitrogen, #11668027) for an additional 2 days. The neurons were then subjected to immunocytochemistry for tau.

### MAP6 shRNA design and validation

For making pSCV2-Venus-mouse MAP6 shRNA, the target sequences used for MAP6 was 5′-GACCTCAACGAGCCATAAATTCAAGAGATTTATGGCTCGTTGAGGTCTTTTTGGAA-3′. The annealed oligonucleotides were ligated into the BamHI/HindIII-digested pSCV2-Venus vector (a gift from Dr Franck Polleux, University of North Carolina at Chapel Hill). The MAP6 shRNA target 1545-1563 (19 mer). Then the MAP6 shRNA efficiency was validated in hippocampal neurons. Briefly, either 3.6 µg of scramble shRNA or MAP6 shRNA were transfected into hippocampal neurons using Lipofectamine 2000 transfection reagent (Invitrogen, 11668030). Neurons were then collected for protein analysis using western blot.

### Western blotting

The cell lysates were collected based on the protocol mentioned in the previous study (Qiang et al., 2018). In short, western blotting was performed using the Bio-Rad system. In essence, 20 µg of protein was loaded into each of the 10 wells on the gel, and the gel was run under 100 volts for 2 h. Subsequently, the gel was transferred at 30 volts overnight. Following this, the membranes were incubated with primary antibodies specific to tau (from Dr Nicholas M. Kanaan, Department of Translational Neuroscience, College of Human Medicine, Michigan State University, MI, USA; 1:2000), MAP6 (from Dr Annie Andrieux, Université Grenoble Alpes, INSERM, U1216, CNRS, CEA, Grenoble Institut Neurosciences, Grenoble, France; 1:600), tyrosinated-tubulin (Sigma-Aldrich, MAB1864; 1:1000) and GAPDH (Abcam, ab8245; 1:2000), followed by HRP-conjugated secondary antibodies (Qiang et al., 2018).

### Immunocytochemistry

Both hippocampal and SCG neurons were fixed using a co-fixative buffer comprising 4% paraformaldehyde (Sigma-Aldrich, 158127-3KG), 1× PHEM buffer (containing 60 mM PIPES, 25 mM HEPES, 5 mM EGTA and 1 mM MgCl_2_, pH 6.9), 0.2% glutaraldehyde (Electron Microscope Science, 16019), and 0.1% Triton X-100 (Sigma-Aldrich, 122H0766) for 10 min at room temperature, followed by three washes with PBS. Subsequently, immunochemistry was conducted following previously established protocols (Qiang et al., 2018) with primary antibodies specific to tau (as above, 1:2000), SMI312 (Biolegend, 837904, 1:1000), βIII-tubulin (Abcam, ab775, 1:700) and laminin (Invitrogen, MA1-06100, 1:400), along with the corresponding secondary antibodies (Qiang et al., 2018).

### *In utero* electroporation

This methodology was executed following established protocols (Bennison et al., 2023). Briefly, pregnant E15.5 mice were anesthetized, and the uterine horn was exposed. Utilizing a pulled-glass micropipette, 1–2 µl of either scramble or MAP6 shRNA plasmids were injected into the lateral ventricle. Following this, electric pulses (consisting of three pulses at 32 V each) were administered via tweezers-type electrodes placed over the uterine muscle, using the CUY21SC electroporator from Nepa GENE. Subsequently, the uterine horn was carefully repositioned within the abdomen, allowing for uninterrupted embryonic development. Venus fluorescent protein encoded in pSCV2-Venus vector was used to visualize electroporated neurons.

### Immunohistochemistry

The brains of P0 mouse were dissected and fixed in a 4% paraformaldehyde for 24 h at 4°C. Subsequently, the brains were transferred to a 25% sucrose (Sigma-Aldrich, S0389) solution to undergo dehydration and facilitate sinking. Cryosectioning was carried out to obtain brain slices with a thickness of 25 µm, with the brains embedded in the M1 matrix (Epredia, 1310). These brain slices were allowed to air-dry overnight and then stained with DAPI (Invitrogen, D1306, 1:20,000) for 5 min. Finally, the stained brain slices were mounted using fluoro-gel mounting medium (Electron Microscopy Sciences, 1798510) and were ready for imaging.

### Imaging, statistical analysis and data processes

Imaging was conducted utilizing either the Leica True Confocal System SP8 for navigation and *Z*-stack acquisition, or the Zeiss AxioObserver. Quantitative analysis was carried out using either Zeiss blue edition software or Fiji software. All experiments were conducted at least three times, and the error bars reflect independent experiments. The acquired data was recorded in Excel, and subsequent bar graphs were generated using GraphPad. For statistical analysis, a two-tailed unpaired *t*-test was employed to compare the average mean values between two comparison groups, while one-way ANOVA with Dunnett's multiple comparisons test was used when comparing the average mean values across more than two comparison groups. The data are presented as mean±s.e.m., statistical significance is denoted on figures in the following manner: *P<0.05 and ^#^*P*<0.05 (*t*-test but not one-way ANOVA), ‘ns’ represents no significant difference. The figures and graphs were organized using Adobe Photoshop 2023.
